# Exploring the role of ferroptosis in pemphigus: identification of diagnostic markers and regulatory mechanisms

**DOI:** 10.3389/fmed.2025.1615865

**Published:** 2025-06-19

**Authors:** Jing Mao, Jianping Lan, Zheyu Zhuang, Ying Chen, Yushan Ou, Xinhong Su, Xueting Zeng, Fuchen Huang, Zequn Tong, Xiaoqing Lv, Hui Ke, Zhenlan Wu, Ying Zou, Bo Cheng, Chao Ji, Ting Gong

**Affiliations:** 1 Department of Dermatology, The First Affiliated Hospital of Fujian Medical University, Fuzhou, China; 2 Department of Dermatology, Mindong Hospital Affiliated to Fujian Medical University, Ningde, China; 3 Department of Dermatology, Nanchong Central Hospital, The Second Clinical Medical College of North Sichuan Medical College Capital Medical University Affiliated Beijing Anzhen Hospital Nan Chong Hospital, Nanchong, China; 4 Institute of Dermatology, Fujian Medical University, Fuzhou, Fujian, China; 5 Fujian Dermatology and Venereology Research Institute, The First Affiliated Hospital, Fujian Medical University, Fuzhou, Fujian, China; 6 Department of Dermatology, Shanghai Children's Medical Center, Shanghai Jiao Tong University School of Medicine, Shanghai, China

**Keywords:** pemphigus, WGCNA, ferroptosis, immune infiltration, biomarker

## Abstract

**Background:**

Pemphigus is an autoimmune blistering disorder characterized by the loss of cell adhesion in the epidermis. Recent studies have suggested a potential link between ferroptosis, a form of regulated cell death dependent on iron, and various diseases. However, the role of ferroptosis-related genes in pemphigus remains largely unexplored. This study aims to investigate the expression patterns and potential biological functions of ferroptosis-related genes in pemphigus, as well as their regulatory mechanisms.

**Methods:**

To achieve this, skin samples from five pemphigus patients and five healthy controls were collected from Fujian Medical University Union Hospital. Additionally, we processed the GSE53873 microarray dataset, which includes 19 pemphigus samples and 5 controls. Differentially expressed genes (DEGs) were identified using the limma R package, followed by Gene Ontology (GO) and Kyoto Encyclopedia of Genes and Genomes (KEGG) enrichment analyses. Weighted Gene Co-expression Network Analysis (WGCNA) was employed to identify co-expressed gene modules related to pemphigus. Machine learning algorithms such as Least Absolute Shrinkage and Selection Operator (LASSO), Random Forest (RF), and eXtreme Gradient Boosting (XGBoost) were used to select key ferroptosis-related genes. Immune cell infiltration was assessed using CIBERSORT and single-sample Gene Set Enrichment Analysis (ssGSEA). Finally, experimental validation was conducted through real-time quantitative PCR, transmission electron microscopy, and drug prediction.

**Results:**

Our results identified 1,840 DEGs in pemphigus patients compared to controls, with significant enrichment in pathways such as PI3K-Akt signaling and fatty acid metabolism. Eleven co-expression modules were identified via WGCNA, with the module highlighted in lightcyan color showing the strongest correlation with pemphigus. Machine learning highlighted ACSL4, SAT2, and XBP1 as potential hub genes with high diagnostic value. Immune analysis revealed increased proportions of activated CD8^+^ T cells and natural killer cells in pemphigus patients. Experimental validation confirmed the presence of ferroptosis morphological features in patient samples.

**Conclusion:**

In conclusion, this study elucidates the involvement of ferroptosis-related genes in pemphigus pathogenesis and identifies potential biomarkers for diagnosis. Further research is warranted to explore therapeutic strategies targeting these pathways.

## 1 Introduction

Pemphigus is a group of rare, life-threatening autoimmune blistering diseases that affect both the skin and mucous membranes, clinically characterized by the formation of flaccid blisters and erosions (1, 2). These blisters are prone to rupture, leading to painful erosions that can become infected and significantly impair quality of life (3). A meta-analysis revealed that the overall incidence of pemphigus worldwide is 2.83 cases per million person-years, with the highest incidence observed in South Asia at 4.94 cases per million person-years (4). Despite advances in treatment, pemphigus remains a life-threatening condition with a significant mortality rate, particularly in severe cases that are refractory to conventional therapies (5). Current treatment strategies for pemphigus primarily involve systemic corticosteroids and immunosuppressive agents, which aim to reduce autoantibody production and inflammation (6). Additionally, more recent therapeutic approaches include the use of CD20 monoclonal antibodies, such as rituximab, which target B cells to further mitigate the production of pathogenic autoantibodies (7). However, these treatments are associated with severe side effects, including increased susceptibility to infections and metabolic complications. Moreover, a subset of patients does not respond adequately to these therapies, and some may experience relapses after treatment, underscoring the need for novel therapeutic targets and strategies (8–10).

Ferroptosis is a form of regulated cell death characterized by iron-dependent lipid peroxidation, driven primarily by the inactivation of glutathione peroxidase 4 (GPX4). This inactivation leads to a depletion of glutathione (GSH), thereby reducing antioxidant defenses. The presence of polyunsaturated fatty acid (PUFA)-containing phospholipids, influenced by ACSL4, further enhances lipid peroxidation, resulting in lethal oxidative stress and cell death. This mechanism has recently garnered attention in the context of various diseases, including cancer and neurodegeneration (11, 12). Studies have shown that ferroptosis plays a role in the pathogenesis of several autoimmune diseases, suggesting that it may also be relevant in pemphigus (13, 14). For instance, ferroptosis has been implicated in systemic lupus erythematosus and rheumatoid arthritis, where it contributes to tissue damage and inflammation (15–17). Given the role of oxidative stress and lipid peroxidation in pemphigus, exploring the involvement of ferroptosis in this disease could provide new insights into its pathogenesis and identify potential therapeutic targets (18, 19).

In this study, we aimed to investigate the expression patterns of ferroptosis-related genes in pemphigus and explore their potential biological functions and regulatory mechanisms. We downloaded and processed the GSE53873 microarray dataset, which contains gene expression profiles from 19 peripheral blood samples of pemphigus patients and 5 peripheral blood controls. Differential gene expression analysis was subsequently performed using the limma R package (20). We also conducted weighted gene co-expression network analysis (WGCNA) to identify co-expression modules and explored their association with pemphigus (21). Furthermore, based on model performance assessed through receiver operating characteristic (ROC) curve analysis and residual distribution evaluation, we employed machine learning algorithms, including Least Absolute Shrinkage and Selection Operator (LASSO), random forest (RF), and eXtreme Gradient Boosting (XGBoost), to identify key ferroptosis-related genes (22). To enhance clinical decision-making, we developed a nomogram model that integrates the expression scores of ACSL4, SAT2, and XBP1 for predicting the risk of pemphigus. Furthermore, molecular docking simulations revealed potential binding sites and affinities between candidate drugs and these target genes, providing valuable insights into the development of targeted therapeutic strategies for pemphigus. Finally, we assessed immune cell infiltration using CIBERSORT and single sample gene enrichment analysis (ssGSEA), and performed gene set enrichment analysis (GSEA) to investigate the biological pathways associated with the identified core genes (23).

Our findings revealed that ferroptosis-related genes are differentially expressed in pemphigus and are involved in key biological processes and pathways related to the disease. We identified 11 co-expression modules, with the module highlighted in lightcyan color designated as the core module. Machine learning algorithms highlighted several potential key genes, and our analyses of immune cell infiltration and GSEA provided further insights into the disease’s immunopathogenesis. Experimental validation confirmed the aberrant expression of ferroptosis-related genes in pemphigus patients, suggesting that targeting ferroptosis could be a promising therapeutic strategy for this devastating disease.

## 2 Materials and methods

### 2.1 Human samples

Skin samples were collected from five pemphigus patients and five healthy control subjects at the First Affiliated Hospital of Fujian Medical University. The research protocol was reviewed and approved by the Ethics Committee of the First Affiliated Hospital of Fujian Medical University (ECFAH of FMU[2015]084–2), and informed consent was obtained from all participants.

### 2.2 Data acquisition and processing

We downloaded the microarray dataset GSE53873, which includes 19 pemphigus and 5 control samples, from the Gene Expression Omnibus (GEO) database^1^ (24). Subsequently, the gene expression matrix was generated, and the probes were annotated in accordance with the annotation file specific to the platform. Gene expression data were normalized using the “normalizeBetweenArrays” function from the limma package in R. A total of 1,539 genes associated with ferroptosis were retrieved from the GeneCards database.^2^

### 2.3 Analysis of differential genes

Differentially expressed genes (DEGs) were identified using the “limma” R package with screening criteria of |log2FC (fold-change)| > 0 and *p*-value < 0.05. The “ggplot2” R package was used to generate a volcano plot of differential genes. Enrichment analysis of Gene Ontology (GO) and Kyoto Encyclopedia of Genes and Genomes (KEGG) was conducted using the “enrichplot” and “GOplot” packages in R. A significance level of *p* < 0.05 was considered indicative of significant enrichment.

### 2.4 WGCNA

The “WGCNA” R package was utilized to identify co-expression gene modules of high biological significance and to investigate the relationship between pemphigus and gene networks. Initially, the top 60% (5,848 genes) with the highest variance values were selected for further analysis. Subsequently, the optimal soft-thresholding power *β* (1–20) was determined using the “pickSoftThreshold” function to establish a scale-free network, with an average connectivity R^2 threshold of 0.85. The adjacency matrix was then transformed into a topological overlap matrix (TOM), and gene ratios and dissimilarities were calculated. Hierarchical clustering combined with the dynamic tree cut function was applied to delineate co-expression modules, requiring each module to comprise at least 50 genes. After merging similar modules, 11 distinct modules were identified, with the lightcyan module designated as the core module. The “ggvenn” package was used to visualize shared genes between two or three groups.

### 2.5 Machine learning

To identify hub ferroptosis genes that significantly contribute to the development of pemphigus, we developed five machine learning models: XGBoost, Support Vector Machine (SVM), LASSO, RF, and Generalized Linear Model (GLM). We utilized the “DALEX” package in R to analyze these models. A ROC curve and residual distribution analysis were performed to determine the optimal model. Ultimately, we employed three distinct machine learning algorithms—LASSO, RF, and XGBoost—to screen for potential key genes associated with ferroptosis. The “glmnet” package was utilized for LASSO regression analysis, employing tenfold cross-validation to determine the optimal solution based on the minimum lambda value. The RF analysis was conducted using the “randomForest” package, while the XGBoost analysis was performed with the “xgboost” package.

### 2.6 Nomogram model based on diagnostic biomarkers

Based on the three hub genes (ACSL4, SAT2, and XBP1) identified through machine learning, we employed the “rms” package in R to construct a nomogram model for predicting the risk of pemphigus in patients. The calibration and ROC curves were subsequently utilized to evaluate the reliability of the model’s predictions. Additionally, a decision curve analysis (DCA) was conducted to determine the clinical utility and whether model-based decisions would be beneficial to patients.

### 2.7 The exploration of potential key genes’ expression patterns

The positions of five ferroptosis-related genes on chromosomes were mapped using the “RCircos” package. The expression levels of these genes in both the pemphigus and control groups were visualized with a boxplot. Additionally, Spearman correlation analysis was conducted to evaluate the correlations among the five expressed ferroptosis-related genes, utilizing the “circlize” and “corrplot” R packages. To assess the diagnostic precision of potential biomarkers, the area under the curve (AUC) score of the ROC curve was computed utilizing the “pROC” package. We conducted predictions for miRNAs and lncRNAs associated with the hub genes using the starBase website.

### 2.8 Evaluation of immune cell infiltration

Immune cell infiltration in pemphigus and normal samples was analyzed using CIBERSORT and ssGSEA. The CIBERSORT algorithm was utilized to calculate the abundance of 18 different types of immune cells within the two groups, and the immune cell composition was illustrated using boxplots. The ssGSEA method was applied, and Wilcoxon test analysis was performed to assess the differences in immune cell expression levels between the two groups, with statistical significance set at *p* < 0.05. Additionally, the “GSVA” and “ggcorrplot” packages were employed to analyze the correlations among various immune cells, using Spearman correlation analysis.

### 2.9 Gene set enrichment analysis

To investigate the biological functions and regulatory pathways related to the three hub genes, single-gene GSEA analysis was conducted using the “limma” and “enrichplot” R packages. The *p*-value <0.05 was regarded as statistically significant, and the most enriched pathways were chosen for visualization.

### 2.10 Real-time quantitative polymerase chain reaction

Total RNA was extracted from human skin tissue samples, including those from three pemphigus patients and three healthy controls, adhering to the protocol specified by the Invitrogen TRIzol reagent (Thermo Fisher Scientific, USA). Following the RNA extraction, cDNA was synthesized using the Invitrogen First-Strand Synthesis System (Thermo Fisher Scientific, USA). Quantitative reverse transcription PCR (qRT-PCR) was performed with the ReverTra Ace qPCR RT Kit (TOYOBO, Japan) on the ABI PRISM 7500 system (Applied Biosystems, USA). GAPDH was utilized as the internal control gene, and the relative expression levels of mRNA were quantified using the 2^^-ΔΔCT^ method. The primer sequences used are detailed in Table 1.

**Table 1:** The primer sequences for qRT-PCR used in this study.

Gene symbol	Forward primer (5′-3′)	Reverse primer (5′-3′)
GAPDH	GGAAGCTTGTCATCAATGGAAATC	TGATGACCCTTTTGGCTCCC
ACSL4	TCCTCCAAGTAGACCAACGCC	GGTCAGAGAGTGTAAGCGGAGAA
SAT2	TGGCTTTGGAGACAATCCTTTC	AGATAAATGGTGCGTCCCTTCC
XBP1	ATGGATTCTGGCGGTATTGACT	AGAGAAAGGGAGGCTGGTAAGG

### 2.11 Transmission electron microscopy

Samples from two pemphigus patients and two healthy controls were fixed in 5% glutaraldehyde, followed by treatment with a potassium ferrocyanide-osmium fixative. Following this, the samples were sectioned and embedded in epoxy resin (Glycidether 100, Germany) for further analysis. Post-staining was carried out using 5% uranyl acetate and 5% lead citrate. The assessment of mitochondrial morphology was performed utilizing a transmission electron microscope (JEM 1010, Tokyo, Japan), with the analysis conducted by a blinded pathologist.

### 2.12 Drug prediction and interaction network

The prediction of molecular compounds or potential drugs targeting hub genes associated with ferroptosis was conducted using the Enrichr platform.^3^ This resource facilitates the collection and integration of extensive gene function information, enabling the discovery of possible targeted drugs for sensitive genomes based on the Drug Signatures database (DSigDB) (25). Furthermore, KEGG enrichment analysis was performed to establish a pathway-target and gene-drug interaction network, which was visualized using Cytoscape.

### 2.13 Analysis of molecular docking

To analyze the regulatory interactions between candidate drugs and core targets, we obtained the molecular structures of ACSL4, SAT2, and XBP1 from the Uniprot database.^4^ The 3D structures of the small-molecule drugs, except for Lead dichloride, were downloaded from the PubChem database.^5^ Additionally, molecular docking and visualization of the results were performed using the CB-Dock website.^6^

### 2.14 Statistical analysis

Statistical analyses were conducted using R software (version 4.3.3) and GraphPad Prism 10 (GraphPad Software, USA). Comparisons between different groups were made using either the unpaired t-test or the Wilcoxon test. Statistical significance was defined at *p < 0.05, **p < 0.01, and ***p < 0.001.

## 3 Results

### 3.1 DEGs and GO/KEGG enrichment analysis

**Figure 1:**
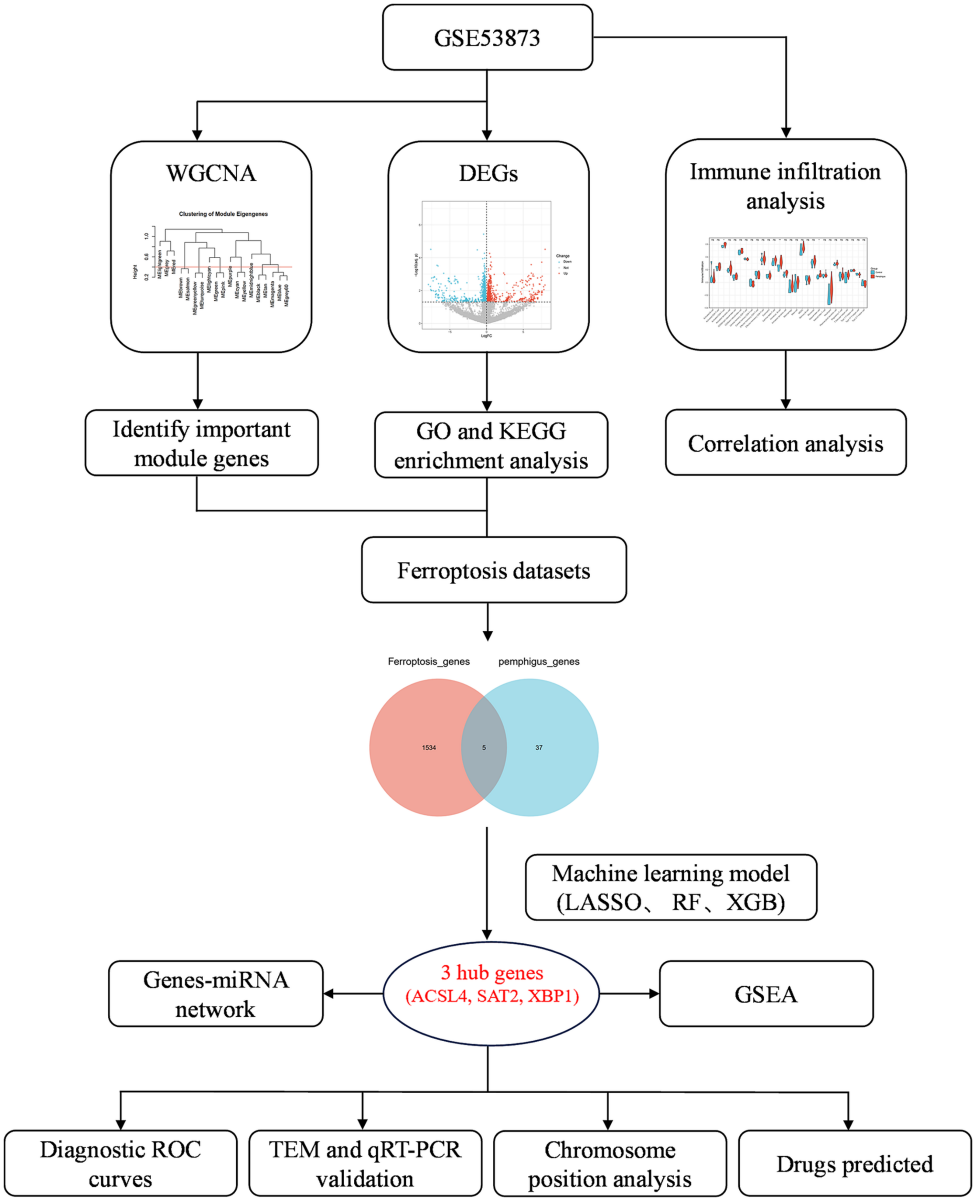
Flowchart depicting the research design, and analysis process of this study.

**Figure 2:**
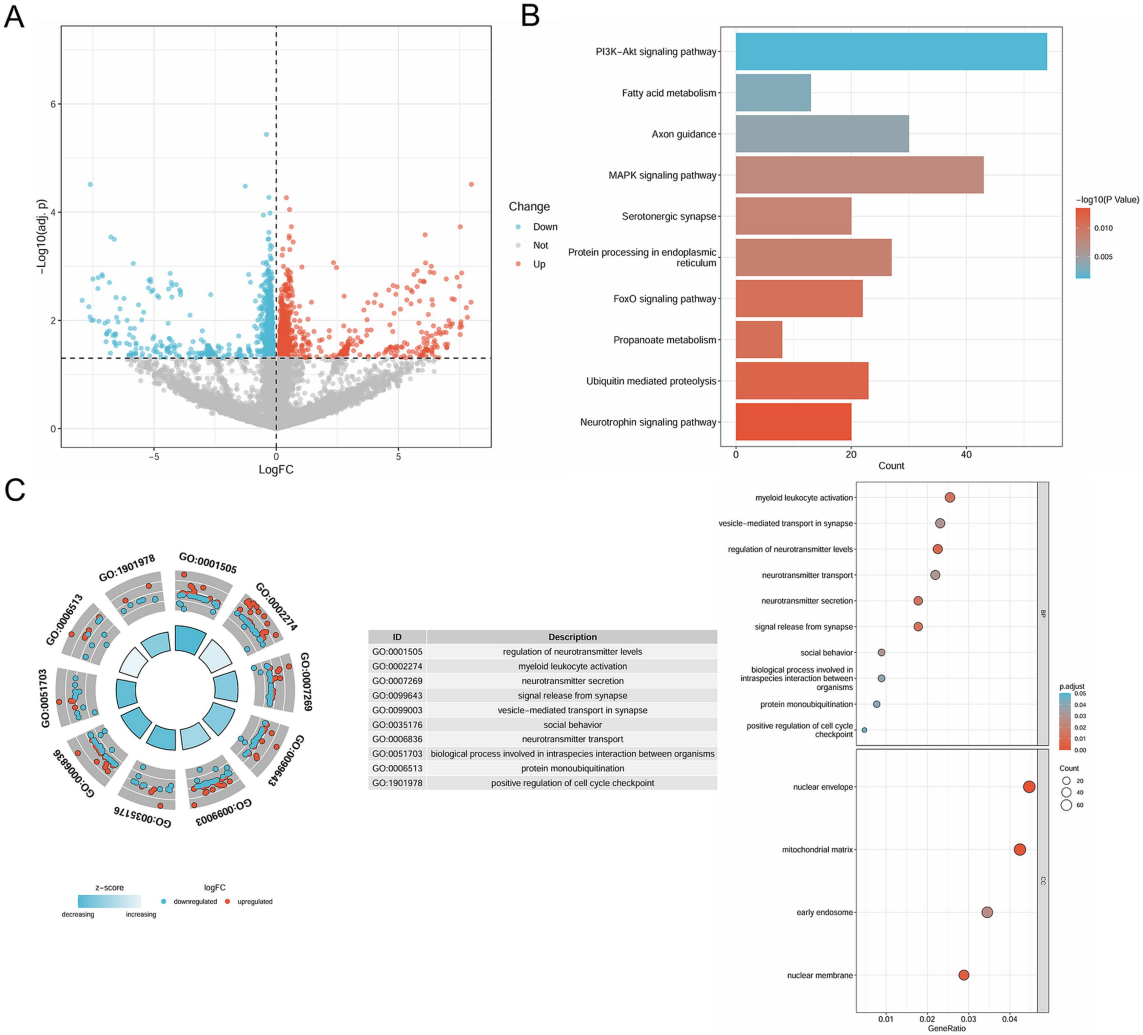
Identification of DEGs in pemphigus and functional enrichment analysis. **(A)** The volcano plot depicts 1840 DEGs between pemphigus and control samples in the GSE53873 dataset. **(B)** KEGG pathway analysis was conducted to explore the enrichment of these DEGs. **(C)** Additionally, GO analysis was performed for functional annotation of the DEGs. DEGs, differentially expressed genes; KEGG, Kyoto Encyclopedia of Genes and Genomes; GO, Gene Ontology.

The research process for this study is illustrated in Figure 1. A total of 1,840 DEGs were identified, comprising 743 upregulated genes and 1,097 downregulated genes in pemphigus patients compared to the healthy control group. The volcano plot (Figure 2A) was utilized to visually depict the genes that exhibited significant differences in expression levels between the pemphigus and control groups. To facilitate understanding of the biological functions associated with the DEGs, KEGG and GO enrichment analyses were conducted. The KEGG enrichment analysis indicated that the DEGs were mainly associated with pathways such as the Pl3K-Akt signaling pathway, Fatty acid metabolism, Axon guidance, MAPK signaling pathway, Serotonergic synapse, Protein processing in endoplasmic reticulum, FoxO signaling pathway, Propanoate metabolism, Ubiquitin mediated proteolysis, and Neurotrophin signaling pathway (Figure 2B). Furthermore, results from the GO analysis revealed that these DEGs were linked to various biological processes, including myeloid leukocyte activation, vesicle-mediated transport in synapse, regulation of neurotransmitter levels, neurotransmitter transport, neurotransmitter secretion, signal release from synapse, nuclear envelope, mitochondrial matrix, early endosome, and nuclear membrane (Figure 2C).

### 3.2 Identification of key modules by WGCNA

**Figure 3:**
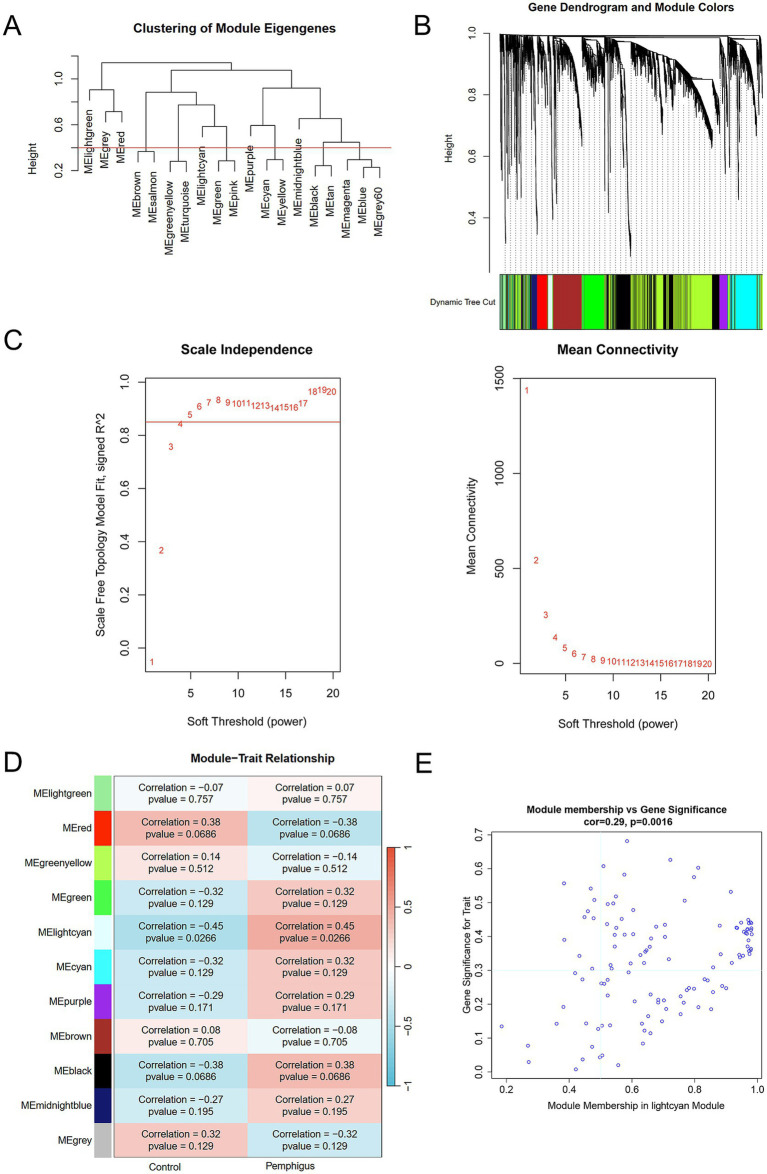
WGCNA identifies key module genes via network construction and module analysis. **(A)** Dendrogram of gene clustering and consensus module eigengenes. **(B)** Color-coded dendrograms from average linkage hierarchical clustering visualize the co-expression modules. **(C)** Network topology analysis across various soft thresholds (*β*). **(D)** The heatmap displays the correlation and *p*-value between module eigengenes and clinical traits in pemphigus. **(E)** Correlation scatterplots illustrate the relationship between module membership and gene significance in lightcyan module. WGCNA, weighted gene co-expression network analysis.

**Figure 4:**
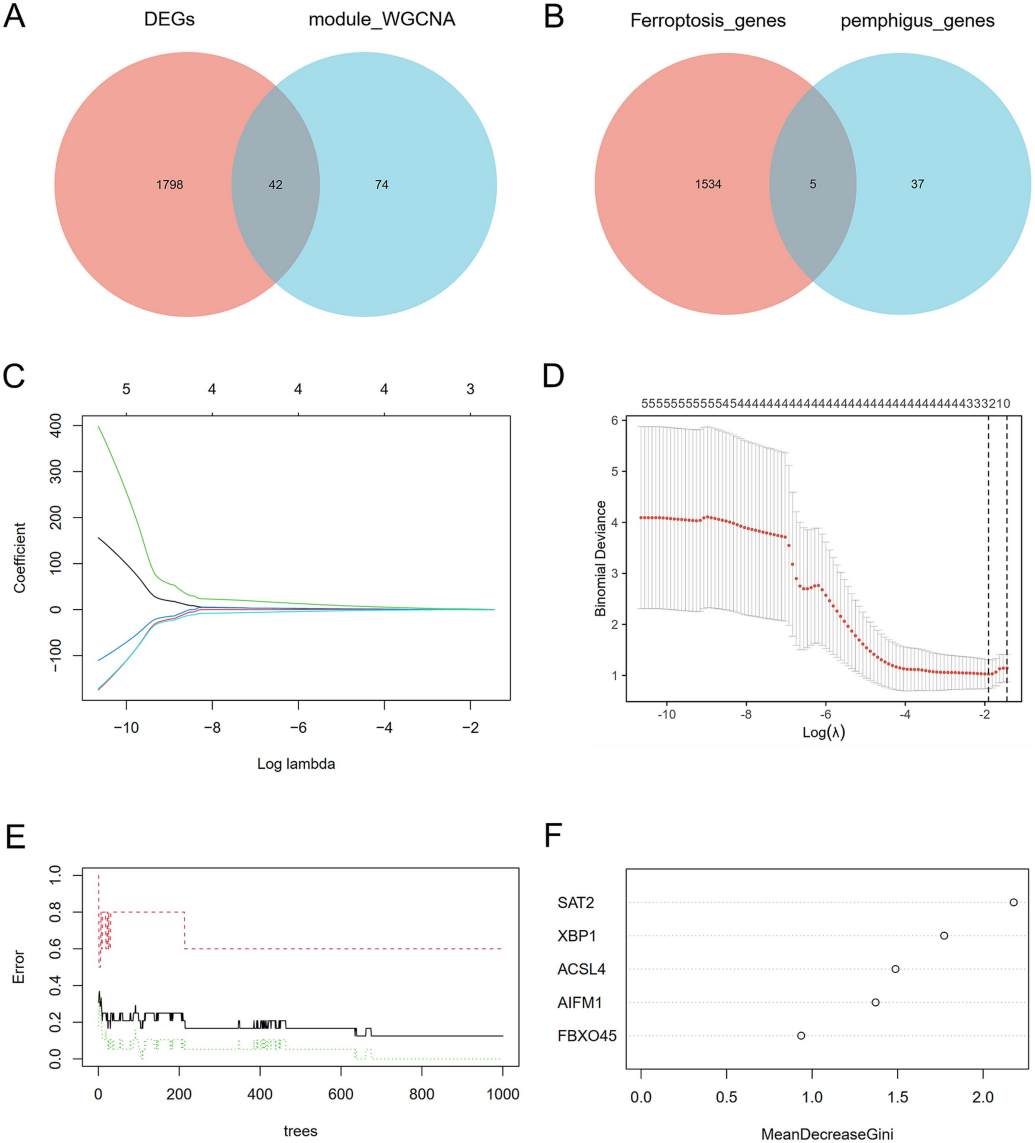
Discovery of potential key genes linked to ferroptosis. **(A)** Venn diagram depicting the overlap between DEGs in pemphigus and genes in the key WGCNA module. **(B)** Venn diagram indicating that 5 genes are identified from the intersection of genes in the ferroptosis datasets and the key pemphigus genes. **(C,D)** LASSO regression analysis identified biomarkers by screening gene numbers and profiling variable coefficients. **(E,F)** The Random Forest model ranked genes according to their importance scores for biomarker screening. LASSO, Least Absolute Shrinkage and Selection Operator.

To identify the most correlated gene modules in pemphigus, WGCNA was conducted. The optimal soft-thresholding power (*β*) was determined to be 6 (Figures 3A–C). A total of 11 modules were identified, with the module highlighted in lightcyan color demonstrating the strongest positive correlation with pemphigus (correlation coefficient = 0.45, *p* = 0.0266) and consisting of 116 genes, making it the focus of further investigation (Figure 3D). Furthermore, a scatter plot was used to illustrate the relationship between gene significance and module membership in the lightcyan module of pemphigus (Figure 3E). A Venn diagram was also created to analyze the overlap between DEGs and genes in the lightcyan module, revealing 42 common genes (Figure 4A).

### 3.3 Machine learning-based discovery of biomarkers

**Figure 5:**
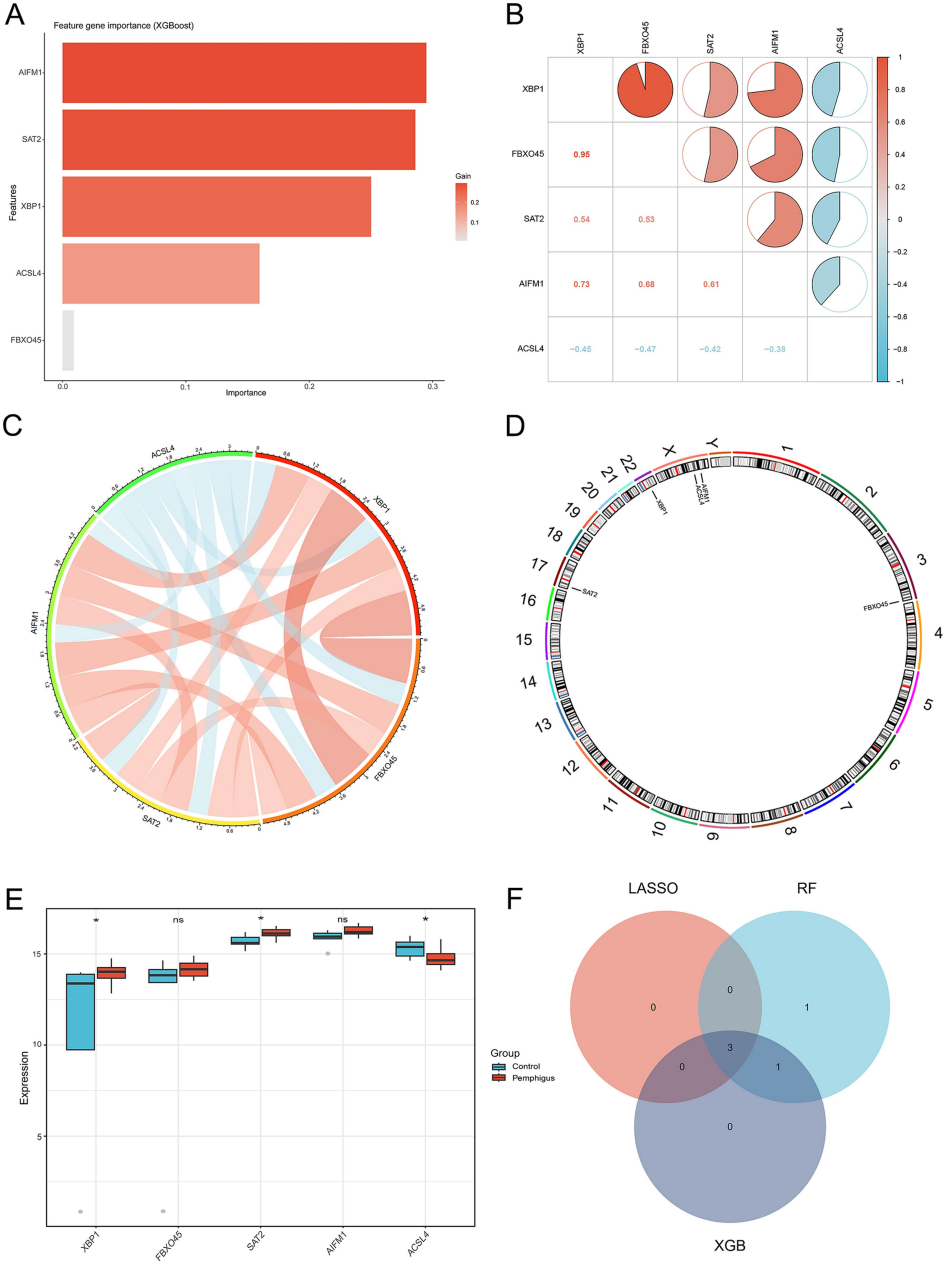
Identification of the 3 candidate hub genes. **(A)** The XGBoost algorithm was used to identify potential biomarkers. **(B,C)** The Spearman correlation analysis was conducted to assess the relationships among ferroptosis-related genes in pemphigus samples. **(D)** The positions of 5 key ferroptosis-related genes on the chromosome. **(E)** Expression analysis of the 5 key ferroptosis-related genes in the pemphigus and control groups. **(F)** A Venn diagram illustrates the overlapping hub genes identified by three distinct machine learning algorithms. *p < 0.05. XGBoost, eXtreme Gradient Boosting.

In order to gain a more comprehensive understanding of the pathogenesis underlying ferroptosis and pemphigus, we identified five intersecting genes by comparing the 1,539 genes from ferroptosis datasets with the 42 pivotal pemphigus genes identified through WGCNA (Figure 4B). Based on ROC curve and residual distribution analysis, the LASSO, RF, and XGBoost algorithms exhibited smaller residual values and higher AUC values (Supplementary Figure S1). Subsequently, three distinct machine learning algorithms were employed to evaluate potential candidate biomarkers based on these five overlapping genes. The LASSO regression model selected three genes (Figures 4C,D), the RF model identified five genes (Figures 4E,F), and the XGBoost model selected four genes (Figure 5A).

### 3.4 Identification of the 3 candidate hub genes

The analysis of the correlation among five potential candidate biomarkers revealed strong interrelationships among these regulators (Figures 5B,C). Notably, FBXO45 and XBP1 exhibited the highest positive correlation, with a correlation coefficient of 0.95. In contrast, ACSL4 showed a negative correlation with XBP1, with a coefficient of 0.45. The positions of the 5 key ferroptosis-related genes were mapped on chromosomes, as shown in Figure 5D. Differential expression analysis was conducted to investigate the expression patterns of the five genes (XBP1, FBXO45, SAT2, AIFM1, and ACSL4) and eight other DEGs associated with ferroptosis (G3BP1, WBP11, TET2, IRF8, FASN, GDPD5, H1-4, and HUWE1) in both the pemphigus and control groups. Results revealed that SAT2 and XBP1 were up-regulated in the pemphigus group compared to the control group, whereas ACSL4 expression was reduced in the pemphigus group (Figure 5E). No significant differences were observed between the two groups for the other genes (Supplementary Figure S2). The three machine learning algorithms collectively identified three overlapping genes, as illustrated in the Venn diagram (Figure 5F). Ultimately, ACSL4, SAT2, and XBP1 were confirmed as candidate hub genes through this analysis.

### 3.5 Diagnostic value of hub genes and lncRNA-miRNA-mRNA network

**Figure 6:**
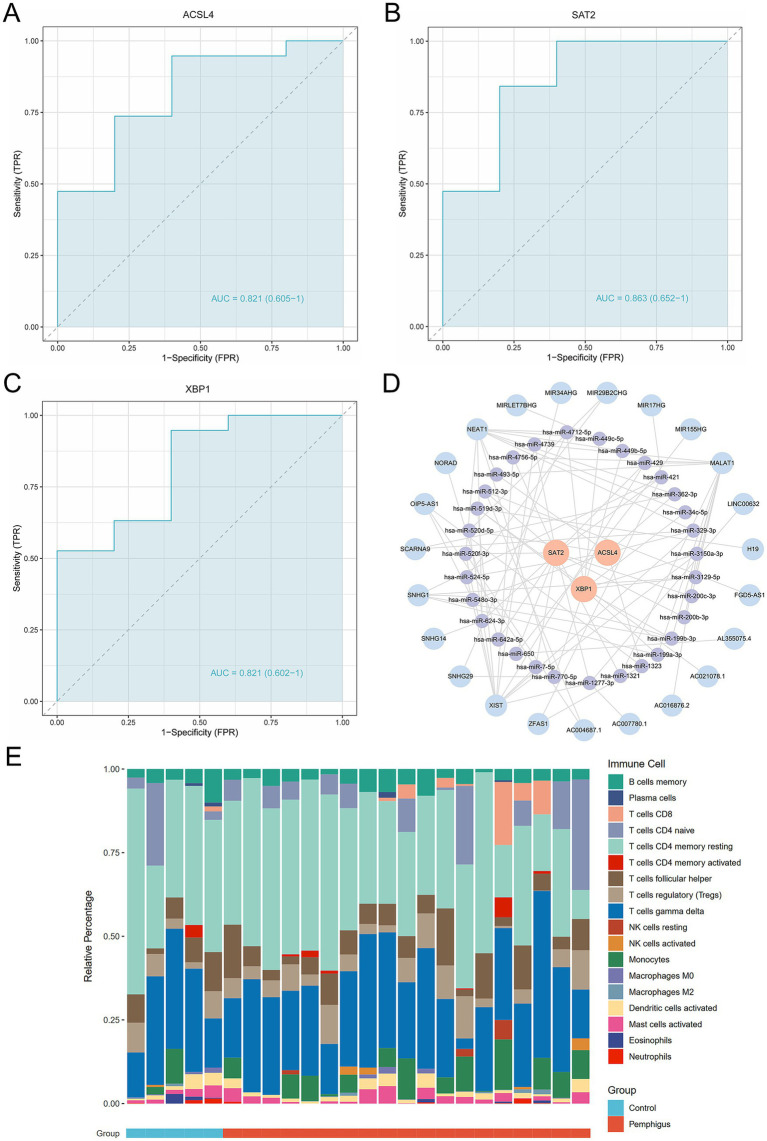
Evaluation of the diagnostic value of the candidate hub genes as biomarkers. **(A–C)** ROC curve analysis of hub genes, including diagnostic markers ACSL4, SAT2, and XBP1. **(D)** Ferroptosis-related mRNA-miRNA-lncRNA regulatory networks in pemphigus. **(E)** The stacked bar chart illustrates the proportions of 18 immune cell types in the pemphigus and control groups. ROC, receiver operating characteristic.

To evaluate the diagnostic potential of ACSL4, SAT2, and XBP1 as biomarkers for pemphigus, ROC curve analyses were performed. The results revealed robust diagnostic efficacy across all three genes, with ACSL4 demonstrating an AUC of 0.821 (95% CI: 0.605–1), SAT2 showing an AUC of 0.863 (95% CI: 0.652–1), and XBP1 yielding an AUC of 0.821 (95% CI: 0.602–1) (Figures 6A–C). These AUC values were all higher than those observed for eight other genes of ferroptosis (Supplementary Figure S2). Collectively, these findings indicate that ACSL4, SAT2, and XBP1 exhibit consistent and significant diagnostic value, supporting their candidacy as promising diagnostic biomarkers for pemphigus. Moreover, we conducted predictions for miRNAs and lncRNAs associated with the three hub genes (ACSL4, SAT2, and XBP1), identifying a total of 31 miRNAs and 23 lncRNAs. These data were subsequently imported into Cytoscape software to construct the ceRNA regulatory network (Figure 6D). This regulatory network illustrates the possible mechanisms regulating ferroptosis in pemphigus.

### 3.6 Construction of nomogram model

We developed a nomogram model incorporating the scores of three hub genes (ACSL4, SAT2, and XBP1) (Supplementary Figure S3). The bias-corrected curve showed a relatively close alignment with the ideal calibration curve, indicating good model calibration. The DCA revealed that within a threshold range of 0.1–1.0, patients using this model experienced greater benefits compared to those with no intervention or complete intervention. Further evaluation via the clinical impact curve indicated that, at a high risk threshold of 0.2–1, the “high risk number” curve closely matched the “high risk with event number” curve, demonstrating the strong predictive capability of the nomogram model. Finally, the area under the ROC curve was 0.989, reflecting well discriminative ability and accuracy in predicting pemphigus.

### 3.7 Immune cell infiltration analysis

**Figure 7:**
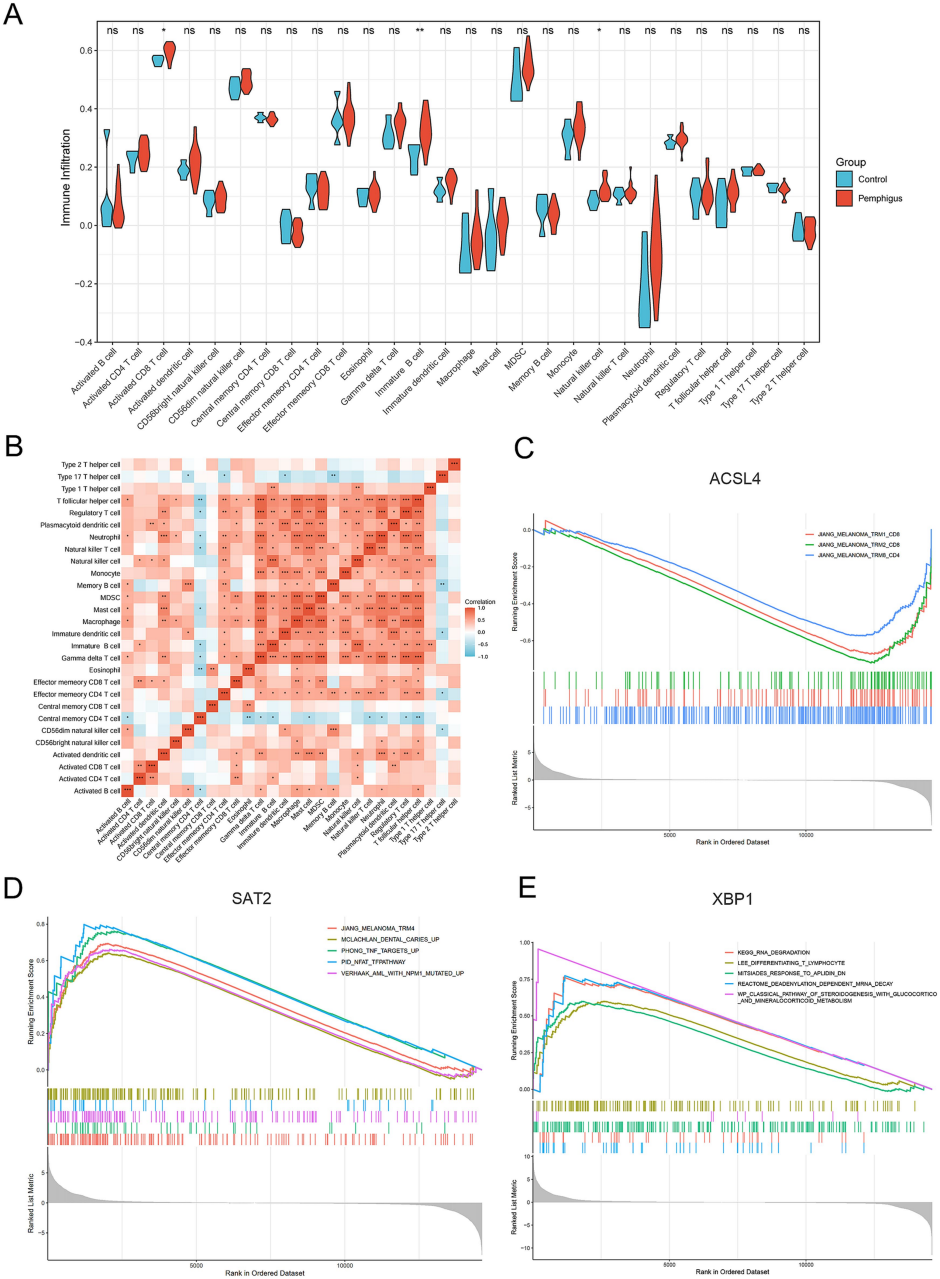
Immune infiltration and gene set enrichment analyses. **(A)** The violin plot illustrates the differences in immune cell types and infiltration between the pemphigus and control groups. **(B)** The heatmap displays the correlations among the proportions of infiltrating immune cell types. **(C–E)** Gene Set Enrichment Analysis of the hub genes. *p < 0.05, **p < 0.01, ***p < 0.001.

To investigate the immune response characteristic of pemphigus, a severe autoimmune disease, we analyzed the proportion of immune infiltrating cells between the pemphigus and control groups (Figure 6E). As shown in Figure 7A, the pemphigus group exhibited a higher prevalence of Activated CD8^+^ T cells, immature B cells, and Natural Killer cells compared to the control group, as illustrated by the violin plot. Furthermore, we performed a correlation heatmap analysis on individual immune cells, revealing significant associations among various immune cell types in the pemphigus samples (Figure 7B). Our results indicated that Activated B cells had positive correlations with Macrophages (*r* = 0.56), T follicular helper cells (*r* = 0.55), and CD56dim natural killer cells (*r* = 0.52). Conversely, Type 17 T helper cells exhibited negative correlations with Memory B cells (*r* = −0.58) and immature dendritic cells (*r* = −0.53). Interestingly, γδ T cells showed a strong positive correlation with myeloid-derived suppressor cells (MDSCs) (*r* = 0.93) and T follicular helper cells (*r* = 0.89). Additionally, Regulatory T cells were positively correlated with γδ T cells (*r* = 0.89) and Neutrophils (*r* = 0.88). These findings suggest unique immune patterns in patients when compared to controls, along with interactions among different types of immune cells.

### 3.8 Gene set enrichment analysis

In our study, we performed a single-gene GSEA to investigate the biological functions and pathways related to key genes involved in ferroptosis, a type of programmed cell death. Our findings revealed a negative correlation between the melanoma and tissue-resident memory T cell (TRM) pathway and the gene ACSL4 (Figure 7C). Additionally, the study highlighted that the gene SAT2 is primarily implicated in pathways that are upregulated in response to tumor necrosis factor (TNF) and the nuclear factor of activated T cells (NFAT), which play crucial roles in the immune response (Figure 7D). Furthermore, the XBP1 gene was observed to be positively associated with several pathways, including those involved in T lymphocyte differentiation, a pathway characterized by mRNA degradation through deadenylation, and a biological pathway critical for steroid hormone production, such as glucocorticoids and mineralocorticoids, which are part of the classical steroidogenesis pathway (Figure 7E).

### 3.9 Experimental validation

**Figure 8:**
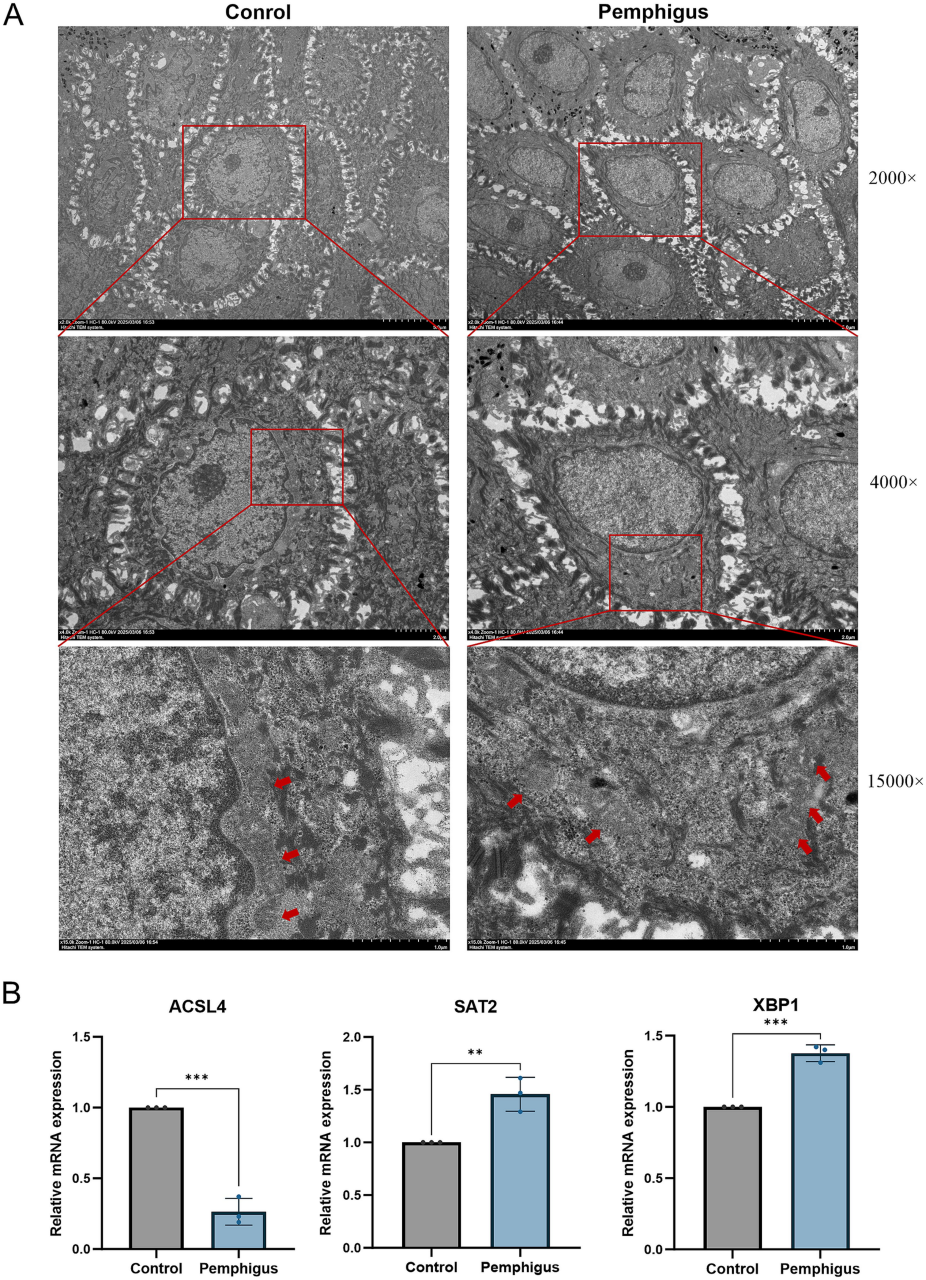
Validation by TEM and qRT-PCR experiments. **(A)** Samples were collected from the roof of bullae skin of pemphigus patients and normal skin adjacent to pigmented nevi for electron microscopy. Compared to the mitochondria in the control group, TEM reveals the presence of reduced mitochondria in keratinocytes, accompanied by a loss of cristae and disruption of the outer mitochondrial membrane. The red arrows indicate the mitochondria, while the values on the right represent different magnification levels. **(B)** qRT-PCR was performed to analyze the expression of hub genes ACSL4, SAT2, and XBP1 in both pemphigus and healthy skin tissues. Data were expressed as mean values with standard deviation (SD). Differences of results were analyzed using unpaired t test. TEM, Transmission electron microscopy; qRT-PCR, quantitative reverse transcription polymerase chain reaction. **p < 0.01, ***p < 0.001.

Further validation using transmission electron microscopy (TEM) on both healthy human skin and pemphigus samples confirmed the morphological characteristics of ferroptosis (Figure 8A). The TEM images showed that the outer mitochondrial membrane was compromised, with a reduction or complete loss of cristae observed in the pemphigus samples. Additionally, the mitochondria appeared smaller, and the density of the mitochondrial membrane was increased. qRT-PCR was employed to evaluate and compare the expression of hub genes in skin tissues from pemphigus patients and healthy controls (Figure 8B). The results showed that SAT2 (*p* = 0.0079) and XBP1 (*p* = 0.0004) had significantly higher expression levels in the pemphigus group compared to the control group. In contrast, the expression levels of ACSL4 were significantly reduced (*p* = 0.0002). These findings corroborated the results of the transcriptomic bioinformatics analysis.

### 3.10 Identification of drug candidates and interaction network

**Figure 9:**
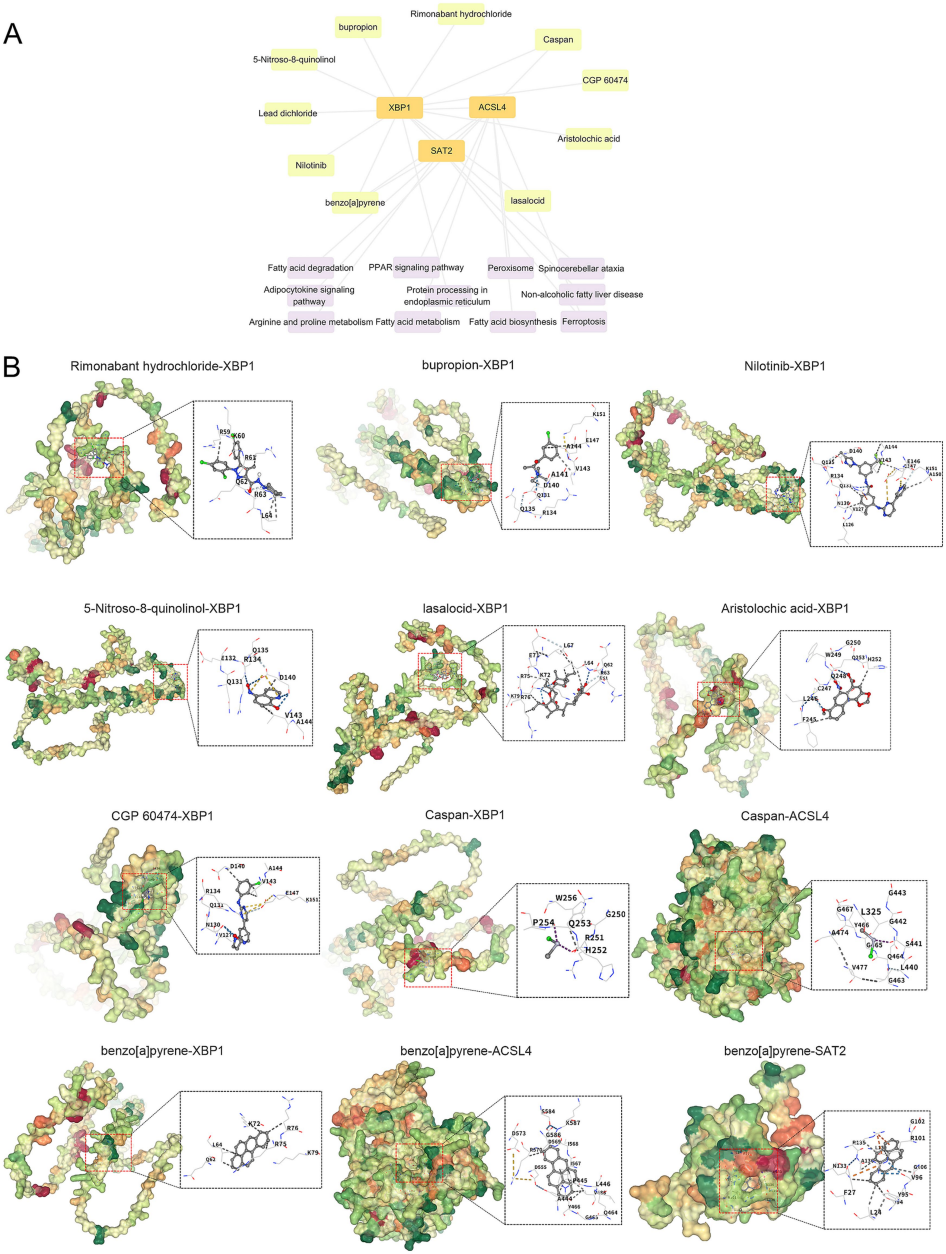
Interaction network and molecular docking results. **(A)** Gene-drug interaction and pathway-target network diagram. **(B)** Molecule docking diagram of candidate drug compounds and core targets in the treatment of pemphigus. The blue dashed line represents a hydrogen bond, while the light blue dashed line indicates a weak hydrogen bond. The gray dashed line signifies a hydrophobic interaction, the bright green line denotes a halogen bond, the yellow line represents an ionic interaction, the orange line indicates a cation-pi interaction, and the dark green line signifies pi-pi stacking.

We conducted a search for potential therapeutic agents targeting key genes ACSL4, SAT2, and XBP1 using the DSigDB database through the Enrichr platform. This search revealed a total of 170 gene-drug interactions. The primary 10 chemical compounds were selected based on their significance, as determined by *p*-values and adjusted p-values. The identified potential drug molecules included Rimonabant hydrochloride (CTD 00003133), bupropion (CTD 00007131), Nilotinib (CTD 00004428), 5-Nitroso-8-quinolinol (CTD 00004584), lasalocid (PC3 UP), Aristolochic acid (CTD 00000005), CGP 60474 (TTD 00002787), Caspan (CTD 00000180), Lead dichloride (CTD 00001407), and benzo[a]pyrene (CTD 00005488) (Table 2). A KEGG functional enrichment analysis revealed that the hub genes are primarily associated with the following functions: ferroptosis, fatty acid biosynthesis, fatty acid degradation, arginine and proline metabolism, the PPAR signaling pathway, protein processing in the endoplasmic reticulum, and other pathways. In the interaction network, drugs or compounds may influence disease outcomes by targeting these hub genes and modulating the corresponding pathways (Figure 9A).

**Table 2:** Candidate drug compounds predicted using DSigDB.

Term	P-value	Adjusted P-value	Combined Score	Genes
Rimonabant hydrochloride CTD 00003133	0.002398	0.047763	4018.416	XBP1
bupropion CTD 00007131	0.002548	0.047763	3729.253	XBP1
Nilotinib CTD 00004428	0.002698	0.047763	3476.150	XBP1
5-Nitroso-8-quinolinol CTD 00004584	0.002847	0.047763	3252.888	XBP1
Lasalocid PC3 UP	0.003745	0.047763	2324.863	XBP1
Aristolochic acid CTD 00000005	0.003895	0.047763	2216.111	XBP1
CGP 60474 TTD 00002787	0.004344	0.047763	1939.487	XBP1
Caspan CTD 00000180	0.004759	0.047763	254.658	XBP1; ACSL4
Lead dichloride CTD 00001407	0.009272	0.047763	764.889	ACSL4
benzo[a]pyrene CTD 00005488	0.010817	0.049701	211519.060	XBP1; ACSL4; SAT2

### 3.11 Molecular docking

Based on the above analysis, nine drug compounds (Rimonabant hydrochloride, bupropion, Nilotinib, 5-Nitroso-8-quinolinol, lasalocid, Aristolochic acid, CGP 60474, Caspan, and benzo[a]pyrene) were selected for molecular docking with the core targets XBP1, ACSL4, and SAT2 (Figure 9B). A lower binding energy between the ligand and receptor indicates a higher likelihood of interaction. With the exception of Caspan, which has binding energies of-1.7 kcal/mol with XBP1 and-2.2 kcal/mol with ACSL4, all other compounds exhibited binding energies lower than-4 kcal/mol.

## 4 Discussion

Pemphigus is a group of rare, autoimmune blistering disorders that affect the skin and mucous membranes. The condition is characterized by the presence of autoantibodies against desmosomal proteins, leading to a loss of cell adhesion and subsequent blister formation (6, 26). The impact of pemphigus on morbidity is significant, as it can cause severe pain, secondary infections, and a substantial reduction in the quality of life, despite being a rare disease. Moreover, the disease often requires long-term immunosuppressive therapy, which carries its own risks and complications (27). The necessity for improved diagnostic and therapeutic strategies is evident, given the profound impact of the disease on patients’ health and daily living (28).

This study aims to explore the expression patterns and potential biological functions of ferroptosis-related genes in pemphigus, utilizing a combination of human sample analysis, bioinformatics, and machine learning techniques. Ferroptosis is a form of regulated cell death characterized by iron-dependent lipid peroxidation, distinct from other forms of cell death like apoptosis or necrosis (29). Given that disruptions in cell death mechanisms are implicated in various autoimmune diseases, understanding the role of ferroptosis in pemphigus could offer novel insights into its pathogenesis (30). This research direction holds promise for developing targeted therapies that could mitigate the disease’s progression and improve patient outcomes (31).

ACSL4, or Acyl-CoA synthetase long-chain family member 4, is an enzyme implicated in the activation of long-chain fatty acids, playing a crucial role in lipid metabolism and ferroptosis (32). Recent studies have highlighted ACSL4’s significant involvement in various cancers and neurodegenerative diseases. For instance, Liu et al. demonstrated that ACSL4 serves as a novel prognostic biomarker in cholangiocarcinoma, correlating with immune cell infiltration and poor prognosis (33). In the context of ferroptosis, ACSL4 has been identified as a key player, with its deletion shown to attenuate lung injury induced by xenobiotic chemicals via suppression of lipid peroxidation (34). SAT2, or Spermidine/Spermine N1-Acetyltransferase Family Member 2, plays a role in the N-acetylation of thialysine and enhances NF-kappaB-dependent transcription. It has been studied in the context of lung adenocarcinoma (LUAD), where it is part of a ferroptosis-related gene signature associated with prognosis (35, 36). SAT1, another family member, is involved in polyamine metabolism and has been implicated in chronic cutaneous inflammation, where it impairs tissue regulatory T cell function, leading to a loss of their suppressive ability and a shift towards a pro-inflammatory phenotype (37). XBP1 (X-box binding protein 1) is a transcription factor that plays a critical role in various cellular processes, including the endoplasmic reticulum stress response, protein secretion, lipid and glucose metabolism, and immune response, making it essential for the development and differentiation of immune cells, particularly in the maturation of B cells into plasma cells (38, 39). Collectively, these findings underscore the multifaceted roles of ACSL4, SAT2, and XBP1 in disease pathogenesis, particularly in ferroptosis and immune modulation, making them promising targets for therapeutic interventions in diseases like pemphigus, where dysregulated cell death and immune responses are pivotal.

Our study identified several key signaling pathways associated with DEGs in pemphigus, notably the PI3K-Akt signaling pathway, fatty acid metabolism, and the MAPK signaling pathway. These pathways are crucial for understanding the pathogenesis and potential therapeutic targets of pemphigus. The PI3K/Akt signaling pathway is linked to the signaling of hormones, growth factors, and nutrients, and plays a crucial role in the growth, proliferation, differentiation, and motility of skin cells (40). Dysregulation of this pathway has been implicated in various autoimmune diseases and cancers (41). In pemphigus, the activation of the PI3K-Akt pathway may disrupt the equilibrium between Th2 and Treg cells, a balance that can be modulated by rapamycin *in vitro* (42). Fatty acids are essential components of cell membranes and are involved in energy production and signaling. Alterations in fatty acid metabolism can affect membrane fluidity and signaling pathways, potentially contributing to the pathogenesis of pemphigus. A study by Wang et al. highlighted the role of gut microbiome-derived metabolites, including short-chain fatty acids, in pemphigus vulgaris, suggesting a link between metabolic changes and disease onset (43). Dysregulated fatty acid metabolism, particularly involving saturated fatty acids, may contribute to the pathogenesis of pemphigus vulgaris by inducing apoptosis and inflammation through the p38 MAPK and NF-kB pathways (44).

The MAPK signaling pathway, particularly the p38 MAPK, has been extensively studied in pemphigus. This pathway is involved in cellular responses to stress and inflammation. Studies have shown that p38 MAPK activation is a downstream event following the loss of intercellular adhesion in pemphigus, contributing to keratinocyte apoptosis and acantholysis (45, 46). Inhibition of p38 MAPK has been shown to block blister formation in pemphigus models, highlighting its potential as a therapeutic target (47, 48). In summary, our findings on the enrichment of these pathways provide insights into the complex molecular mechanisms underlying pemphigus. The PI3K-Akt signaling pathway, fatty acid metabolism, and MAPK signaling pathways all contribute to the disease’s pathogenesis. Understanding these pathways’ roles can help develop targeted therapies to improve disease management and patient outcomes.

Our study provides significant insights into the immune cell infiltration in pemphigus lesions, highlighting the critical role of various immune cells in the pathogenesis of the disease. Previous research has established that pemphigus is characterized by a robust infiltration of immune cells, including B and T lymphocytes, neutrophils, and eosinophils, which contribute to the disease’s pathology (49). Specifically, studies have shown that the lesional skin of pemphigus patients contains substantial numbers of CD4^+^ T helper cells, CD8^+^ cytotoxic T cells, and B cells, which are essential for the autoimmune response against desmoglein, the primary antigen in pemphigus (50). Our findings align with these observations, demonstrating a significant increase in activated CD8^+^ T cells, immature B cells, and natural killer cells in pemphigus lesions compared to healthy controls. The presence of these immune cells suggests a complex interplay between innate and adaptive immune responses in the disease’s progression. Notably, the activation of CD8^+^ T cells and the increased levels of plasma cells and type 1 macrophages (M1) highlight the role of cellular immunity in mediating tissue damage and inflammation in pemphigus (51).

The immune cell infiltration in pemphigus lesions has significant implications for understanding the disease’s pathogenesis and developing targeted therapies. The identification of key immune cells and their functional states provides a basis for exploring novel therapeutic strategies aimed at modulating immune responses.

We also developed a nomogram model integrating the expression scores of ACSL4, SAT2, and XBP1 to predict pemphigus risk, which showed good calibration and significant clinical utility. Additionally, using the Enrichr platform and DSigDB database, we identified 170 gene-drug interactions targeting these hub genes, with the top 10 compounds including Rimonabant hydrochloride, bupropion, and Nilotinib. KEGG enrichment analysis linked these genes to ferroptosis, fatty acid metabolism, and PPAR signaling pathways. Molecular docking simulations further revealed potential binding sites and affinities between these drugs and the hub genes. These findings provide critical insights into drug-target interactions and could guide the development of novel therapeutics targeting ferroptosis-related genes in pemphigus, thereby enhancing clinical decision-making and therapeutic strategies for the disease.

In reflecting upon the limitations of this study, several aspects warrant consideration. Firstly, while the study incorporates wet lab experiments, such as electron microscopy and qRT-PCR, the range of experiments is relatively limited. Secondly, the sample size consists of 19 pemphigus patients and 5 healthy controls, which may still limit the applicability of the findings. Additionally, the lack of extensive clinical validation means that the results have not been confirmed in a clinical setting, which is crucial for translating these findings into potential therapeutic strategies. Moreover, while DEGs have provided valuable insights into the molecular mechanisms of pemphigus, further functional studies are essential to clarify their causal roles and prioritize them for therapeutic intervention. Future research should include gene knockout or overexpression experiments, along with *in vitro* and *in vivo* models, to evaluate the effects of gene manipulation on disease phenotypes and to identify potential therapeutic targets with greater confidence.

## 5 Conclusion

In conclusion, this study successfully identified ferroptosis-related differentially expressed genes and elucidated their potential biological functions in pemphigus. The selection of key genes through WGCNA and machine learning provides a deeper understanding of the disease’s molecular underpinnings. The evaluation of immune cell infiltration and the significant pathways involving core genes further enrich our comprehension of pemphigus pathogenesis. Looking forward, these findings could pave the way for novel therapeutic targets and strategies, although further validation in larger, clinically diverse cohorts and experimental settings is essential to fully realize their potential impact.

## Data Availability

Publicly available datasets were analyzed in this study. This data can be found here: https://www.ncbi.nlm.nih.gov/geo/query/acc.cgi?acc=GSE53873.

## Footnotes

^1^ https://www.ncbi.nlm.nih.gov/geo/

^2^ https://www.genecards.org/

^3^ https://maayanlab.cloud/Enrichr/

^4^ https://www.uniprot.org/

^5^ https://pubchem.ncbi.nlm.nih.gov/

^6^ https://cadd.labshare.cn/cb-dock2/php/blinddock.php
