# Correction: Exploring the role of ferroptosis in pemphigus: identification of diagnostic markers and regulatory mechanisms

**DOI:** 10.3389/fmed.2026.1899701

**Published:** 2026-06-16

**Authors:** Jing Mao, Jianping Lan, Zheyu Zhuang, Ying Chen, Yushan Ou, Xinhong Su, Xueting Zeng, Fuchen Huang, Zequn Tong, Xiaoqing Lv, Hui Ke, Zhenlan Wu, Ying Zou, Bo Cheng, Chao Ji, Ting Gong

**Affiliations:** 1 Department of Dermatology, The First Affiliated Hospital of Fujian Medical University, Fuzhou, China; 2 Department of Dermatology, Mindong Hospital Affiliated to Fujian Medical University, Ningde, China; 3 Department of Dermatology, Nanchong Central Hospital, The Second Clinical Medical College of North Sichuan Medical College Capital Medical University Affiliated Beijing Anzhen Hospital Nan Chong Hospital, Nanchong, China; 4 Institute of Dermatology, Fujian Medical University, Fuzhou, Fujian, China; 5 Fujian Dermatology and Venereology Research Institute, The First Affiliated Hospital, Fujian Medical University, Fuzhou, Fujian, China; 6 Department of Dermatology, Shanghai Children's Medical Center, Shanghai Jiao Tong University School of Medicine, Shanghai, China

**Keywords:** pemphigus, WGCNA, ferroptosis, immune infiltration, biomarker

Affiliation 1 “Department of Dermatology, The First Affiliated Hospital of Fujian Medical University, Fuzhou, China” was omitted for author Ting Gong. This author should have affiliations 1 and 6.

This affiliation has now been added for author Ting Gong.

The original version of this article has been updated.

