# Supplementary material for: Exploring the role of ferroptosis in pemphigus: identification of diagnostic markers and regulatory mechanisms
Source: Front Med (Lausanne). 2025 Jun 19;12:1615865. doi: 10.3389/fmed.2025.1615865 (PMC12221918; doi:10.3389/fmed.2025.1615865)
Supplement: Supplementary file 1 [file Supplementary_file_1.docx]

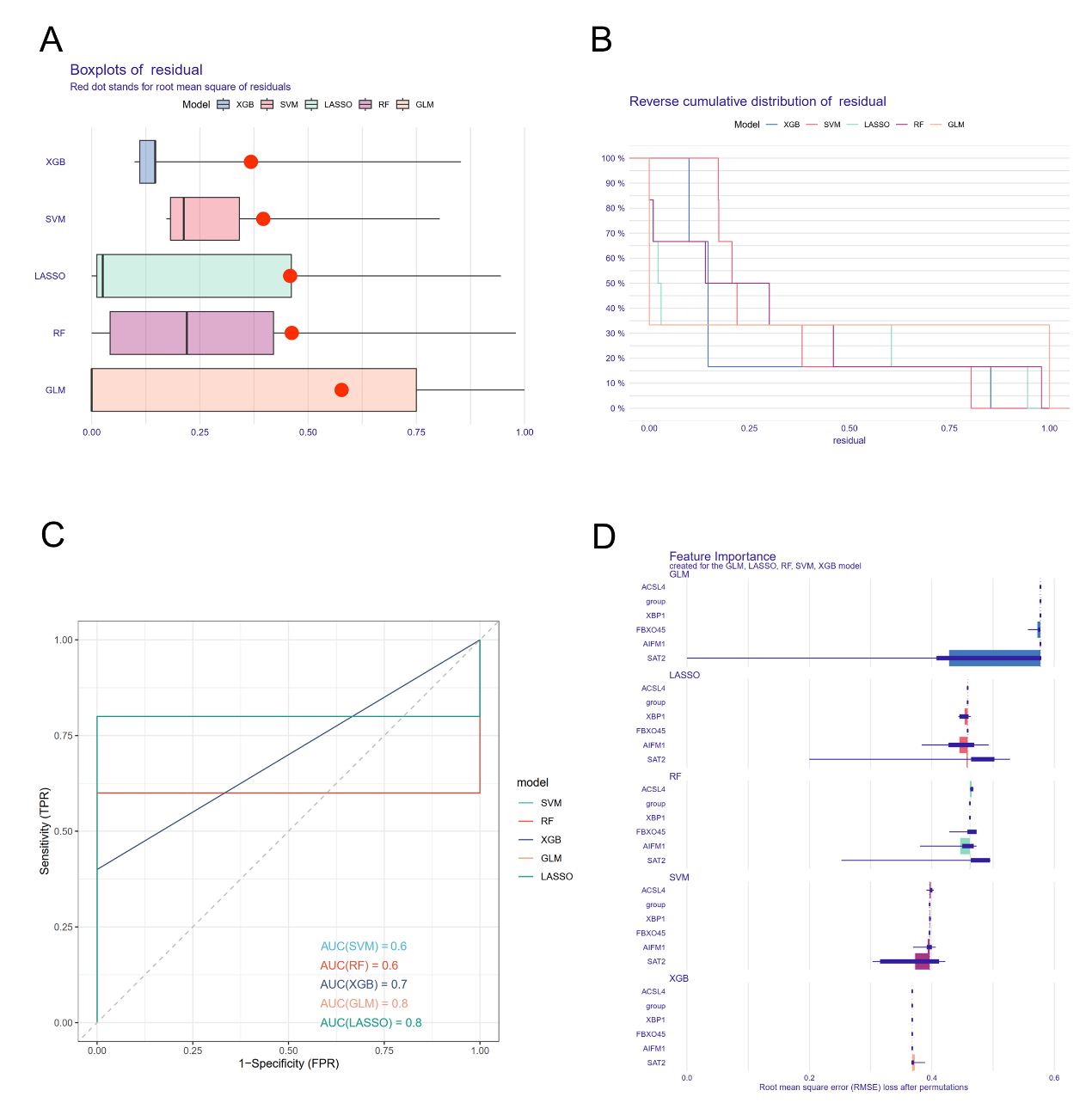


**Supplementary Figure S1** Building and assessing machine learning models for XGBoost, SVM, LASSO, RF, and GLM. (A) Box plot of the residuals for the five machine learning models. (B) Cumulative residual distribution for the five machine learning models. (C) Evaluation of the classification performance of each machine learning model using ROC curves. (D) Assessment of the importance of genes in each machine learning algorithm. SVM, support vector machine; GLM, generalised linear model.
