# Supplementary material for: Exploring the role of ferroptosis in pemphigus: identification of diagnostic markers and regulatory mechanisms
Source: Front Med (Lausanne). 2025 Jun 19;12:1615865. doi: 10.3389/fmed.2025.1615865 (PMC12221918; doi:10.3389/fmed.2025.1615865)
Supplement: Supplementary file 2 [file Supplementary_file_2.docx]

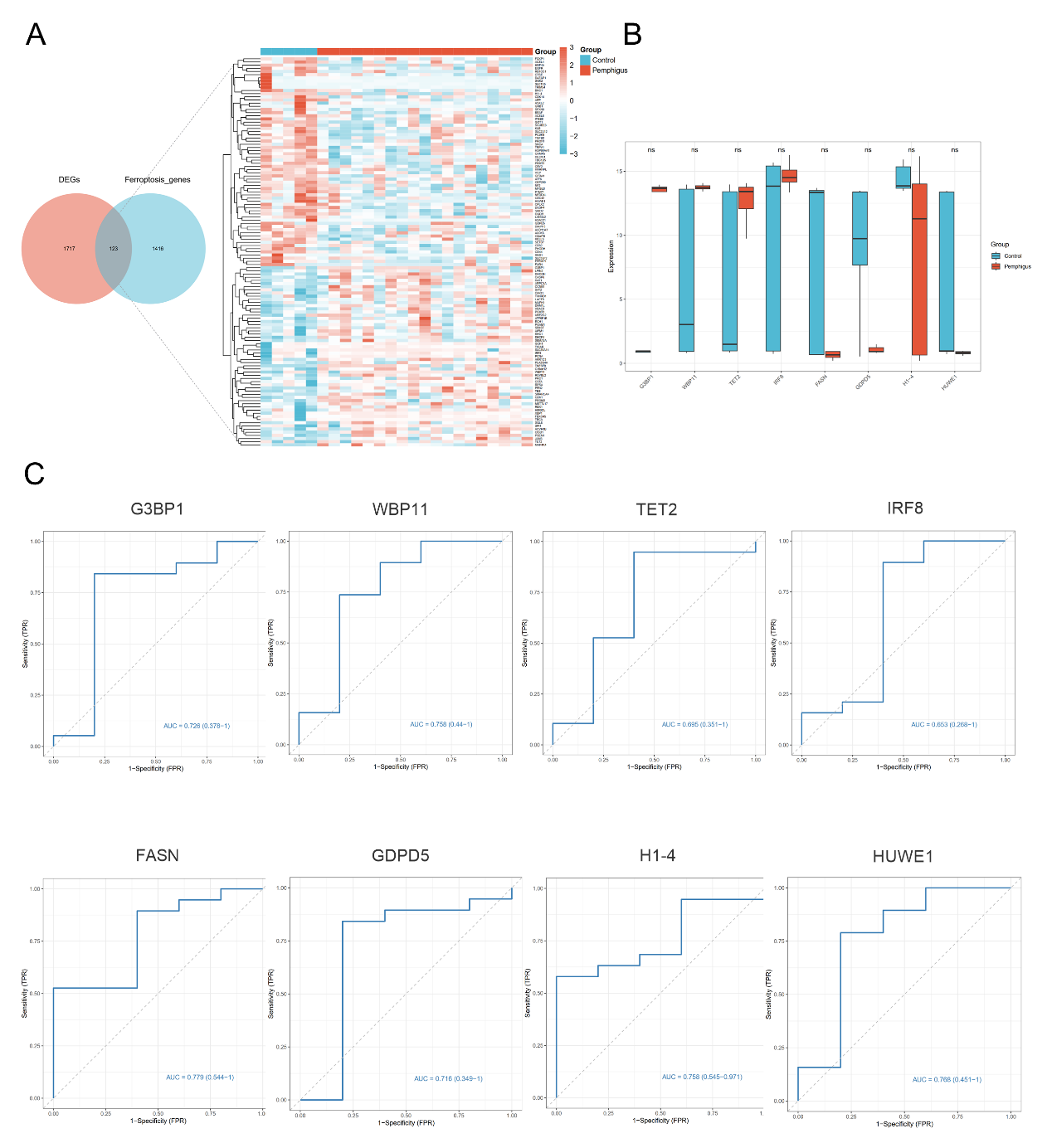


**Supplementary Figure S2** (A) Venn diagram and heatmap illustrating the intersection of differentially expressed genes in pemphigus and ferroptosis datasets. (B) A box plot illustrating the distribution of expression levels for genes involved in ferroptosis. (C) ROC curves for eight diagnostic genes of ferroptosis. Ns, no significance.
