# Supplementary material for: Exploring the role of ferroptosis in pemphigus: identification of diagnostic markers and regulatory mechanisms
Source: Front Med (Lausanne). 2025 Jun 19;12:1615865. doi: 10.3389/fmed.2025.1615865 (PMC12221918; doi:10.3389/fmed.2025.1615865)
Supplement: Supplementary file 3 [file Supplementary_file_3.docx]

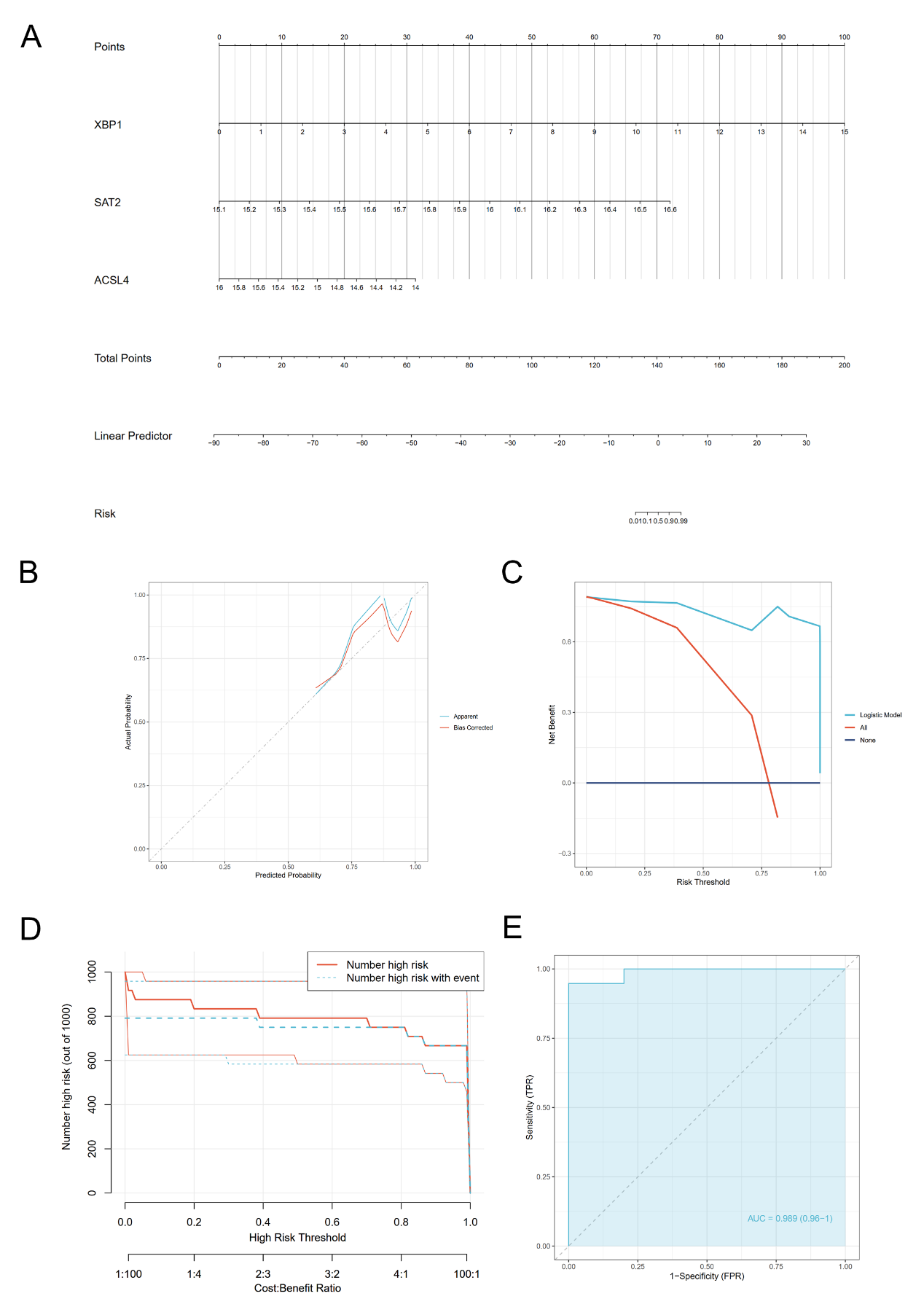


**Supplementary Figure S3** Development and validation of a nomogram model. (A) A nomogram was constructed to estimate the prevalence of pemphigus based on three hub genes. (B) The calibration curve demonstrated the predictive validity of the nomogram model. (C) DCA curve evaluated the clinical applicability of the nomogram model. (D) The clinical impact curve illustrated the effectiveness of the predictive model in a clinical context. (E) The ROC curve demonstrated the predictive accuracy of the nomogram model. DCA, decision curve analysis.
